# Adult Sex‐Ratio Bias Does Not Lead to Detectable Adaptive Offspring Sex Allocation Via Nest‐Site Choice in a Turtle With Temperature‐Dependent Sex Determination

**DOI:** 10.1002/ece3.70543

**Published:** 2024-11-13

**Authors:** Claudia Crowther, Clare I. M. Adams, Andy Fondren, Fredric J. Janzen

**Affiliations:** ^1^ Departments of Fisheries and Wildlife & Integrative Biology, W.K. Kellogg Biological Station Michigan State University Hickory Corners Michigan USA; ^2^ Coastal People Southern Skies Victoria University of Wellington Te Herenga Waka Wellington New Zealand; ^3^ Department of Ecology, Evolution, and Organismal Biology Iowa State University Ames Iowa USA

**Keywords:** climate change, environmental sex determination, freshwater turtles, maternal effects, reproductive behaviour, sex ratio

## Abstract

Sex‐ratio theory predicts that parents can optimise their fitness by producing offspring of the rare sex, yet there is a dearth of empirical evidence for adaptive sex allocation in response to the adult sex ratio (ASR). This is concerning, as anthropogenic disruption of the sex ratios of reproductive individuals threatens to cause demographic collapse in animal populations. Species with environmental sex determination (ESD) are especially at risk but may possess the capacity to adaptively influence offspring sex via control over the developmental environment. For example, reptiles with temperature‐dependent sex determination (TSD) could conceivably choose nest sites with thermal characteristics that produce offspring of the rare sex. To test this hypothesis, we seeded three secure outdoor ponds with different sex ratios (~M:F 3:1, 1:1, and 1:3) of adult painted turtles ( *Chrysemys picta* ), a reptile species with TSD. We then quantified nesting traits that could influence nest temperature and thus offspring sex ratio, including nesting date, nest depth, and nest canopy cover. We found no directional relationship between the ASR treatments and any measured nest traits and thus rejected our hypothesis. Interestingly, increased maternal body size was associated with reduced nest canopy cover, and this trend was more pronounced in the biased ASR treatments. If adaptive sex allocation occurs in this system, it instead may manifest via maternal epigenetic predisposition of offspring sex or in response to a phenomenon other than the ASR.

## Graphical abstract

Sex‐ratio theory predicts that parents can optimise their fitness by producing offspring of the rarer sex, yet there is a dearth of empirical evidence for adaptive sex allocation in response to the adult sex ratio (ASR). We found no evidence that *Chrysemys picta* , a turtle with temperature‐dependent sex determination, influences offspring sex via nesting behaviour in response to ASR bias. Our result is concerning, as it suggests that behavioural responses to anthropogenic disruption of sex ratios may be limited in this species.

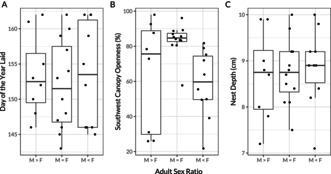

## 1 Introduction

Sex‐ratio theory predicts that parents should bias reproductive effort toward offspring of the sex that will provide the greatest return on this investment, given parental quality and environmental conditions (Trivers and Willard 1973; Werren and Charnov 1978; Fisher 1999). Substantial research effort has been dedicated to investigating sex allocation in response to individual parental traits (Cameron 2004; Rosenfeld and Roberts 2004; Sheldon and West 2004; Brooksmythe et al. 2017; Szász, Garamszegi, and Rosivall 2019). However, we know far less about sex allocation in response population characteristics, such as the adult sex ratio (the sex ratio of breeding‐age individuals, ASR), despite a wealth of conceptual research supporting the idea that parents maximise their fitness when they produce offspring of the rare sex (Frank 1990; Hardy 1997, 2002; Fisher 1999; Schärer 2009; West 2009; Schwanz and Georges 2021; Greeff and Kjellberg 2022; Gardner 2023). Anthropogenic environmental change can influence adult recruitment and survival in a sex‐biased manner (Pritchard and Trebbau 1984; Aresco 2005; Grüebler et al. 2008; Reid and Peery 2014; Refsnider and Janzen 2016; Dufek, Battan‐Horenstein, and Mulieri 2021; Kohno 2021; Roberts et al. 2023; Firman 2024) with the potential to cause demographic decline via reduction in breeding success, declining juvenile recruitment, loss of genetic diversity, and increasing sexual conflict (Solberg et al. 2002; Le Galliard, Fitze, Ferrière, et al. 2005; Grayson et al. 2012; Székely, Weissing, and Komdeur 2014; Morrison et al. 2016; Regan et al. 2020; Chiba et al. 2023). In this context, understanding the propensity of individuals to respond to ASR disturbances is critical to assessing the risk of species extinction.

A diverse range of species adjust offspring sex in response to parental traits (e.g., Sheldon and West 2004; Szász, Garamszegi, and Rosivall 2019; Geffroy and Douhard 2019). When males experience greater reproductive variability than females, mothers in good condition are predicted to bias sex allocation to sons, as sons benefit more from increased resource investment than daughters in these circumstances (Trivers and Willard 1973). Meta‐analyses support a weak effect of maternal condition on offspring sex in a wide range of mammal species and a more substantial effect when inter‐species variability and mechanisms of sex‐ratio manipulation are considered (Cameron 2004; Rosenfeld and Roberts 2004; Sheldon and West 2004). Similarly, when male reproductive success is highly variable and dependent on costly traits, parents are predicted to bias offspring sex ratios in response to male sexually‐selected traits (Burley 1981, 1986). This phenomenon is well studied in birds and to a lesser degree in mammals, and meta‐analyses of these studies have shown a weak relationship between male attractiveness and offspring sex ratios, particularly in cases where researchers examined male traits proven to be preferred by females (Brooksmythe et al. 2017; Szász, Garamszegi, and Rosivall 2019).

In contrast to individual traits, we understand less about adaptive sex allocation in response to population‐level characteristics, such as the ASR, despite the sex‐specific influence of demography on offspring fitness. In populations with biased sex ratios, rare‐sex individuals enjoy greater reproductive success; thus, frequency‐dependent selection favours the production of rare‐sex offspring, stabilising the sex ratio (Fisher 1999). Therefore, selection should favour traits that allow parents to detect ASR bias and adjust offspring sex allocation in response (Werren and Charnov 1978). Several invertebrate species adaptively influence clutch sex ratio in response to ASR (McLain and Marsh 1990; Shuker and West 2004; Brooksmythe et al. 2018). There is mixed evidence from avian species regarding adaptive responses to ASR, with some studies describing a weak to moderate relationship between ASR and offspring sex ratios (Michler et al. 2013; Benvenuti et al. 2018; Arrieta et al. 2024), and others observing no effect (Bensch et al. 1999; Wheelwright and Seabury 2003; Perlut et al. 2014). Additionally, some bird species may respond to sex‐biased mortality by reducing resource allocation to the sex with a higher mortality risk (Heinsohn et al. 2021). In mammals, there is some evidence that offspring sex ratio responds to maternal exposure to males (Hamel, Festa‐Bianchet, and Côté 2016; Firman 2020), but not to other social factors such as population density (Zipple et al. 2023).

In addition to the lack of research on adaptive sex allocation in response to population‐level characteristics, a taxonomic bias is also evident. Parental manipulation of offspring sex is underexplored in reptiles when compared with the extensive literature in mammals and birds (Navara 2018). There is evidence that parental condition and access to resources influence offspring sex ratios in some reptile species (Olsson, Wapstra, and Tobias 2005; Warner, Lovern, and Shine 2007; Navara 2018; Geffroy and Douhard 2019; but see Grüebler et al. 2008). There is mixed evidence for the adaptive benefit of the seasonal variation in sex ratios observed in some reptiles with TSD (Shine 1999; for: Harlow and Taylor 2000; Warner and Shine 2005; against: Uller and Olsson 2006; Pezaro, Thompson, and Doody 2016). Yet, few studies have investigated whether reptiles bias sex allocation in response to ASR. Field observations of alpine skinks suggest that maternal reproductive investment is sex‐biased and negatively associated with ASR in this species (Olsson and Shine 2001). However, two experiments that manipulated ASR in different lizard species found no evidence of biased sex allocation in response to ASR (Le Galliard, Fitze, Cote, et al. 2005; Allsop et al. 2006), and a third study found an effect in the opposite direction than expected (Warner and Shine 2007). To our knowledge, these are the only studies to examine adaptive sex allocation in response to manipulated ASR in reptiles.

Studies of parental adaptive control over offspring sex in reptiles are complicated by aspects of reptile biology such as long lifespan and the variety of sex‐determining mechanisms present in these taxa (Merkling et al. 2018; Navara 2018). However, this variation in sex determination and the simple patterns of parental investment seen in reptiles can make them excellent candidates for exploring mechanisms of adaptive sex allocation (Wapstra et al. 2007). Furthermore, reptiles are experiencing anthropogenic sex‐ratio bias across life stages via climate, pollution, and sex‐biased mortality, which ultimately influence the ASR (Pritchard and Trebbau 1984; Aresco 2005; Reid and Peery 2014; Refsnider and Janzen 2016; Kohno 2021; Roberts et al. 2023). This risk is especially high for species with TSD (Mitchell and Janzen 2010; Valenzuela et al. 2019; Laloë, Schofield, and Hays 2024; but see Hays et al. 2017). It is, therefore, imperative that we understand the capacity and mechanisms for responses to ASR bias in reptiles.

In nest‐building species, parents may respond to environmental change by varying nesting phenology, nest‐site choice, and nest construction in order to provide optimal developmental conditions for their offspring (Mainwaring et al. 2017; Du et al. 2023). For reptiles with TSD, nesting behaviour (e.g., variation in nest phenology, shade cover, depth, moisture, and substrate type) has the capacity to influence nest temperature and, subsequently, offspring sex (St. Juliana, Bowden, and Janzen 2004; Tucker et al. 2008; Telemeco, Elphick, and Shine 2009; Mitchell and Janzen 2010; Refsnider and Janzen 2012; Somaweera and Shine 2013; Bodensteiner et al. 2023). Additionally, nesting behaviour displays heritable variation under some environmental and maternal conditions and thus could evolve in response to selection (Kolbe and Janzen 2002; McGaugh et al. 2010; McGaugh and Janzen 2011; Janzen et al. 2019; Delaney, Hoekstra, and Janzen 2020). Therefore, reptile mothers may respond to environmentally‐induced ASR bias by adjusting nesting behaviour to produce offspring of the rare sex.

However, simply observing that nest‐site choice varies with environmental conditions is not sufficient to confirm that nesting behaviour responds plastically to ASR, as other selective pressures, such as offspring viability, may influence this behaviour (Refsnider and Janzen 2010). In one population of painted turtles (*Chrysemys picta*), nest temperatures differed from co‐located random sites to a degree that influenced offspring sex but not survival, suggesting that nesting behaviour could be responding to sex‐ratio selection (Mitchell, Maciel, and Janzen 2013). Evidence that mothers vary their nesting behaviour in response to the proportion of adult males in their population would confirm parental intent to allocate offspring sex in response to the ASR.

In this investigation, we examine if maternal behaviour with the capacity to influence offspring sex in *C. picta*, a freshwater turtle with TSD, responds to ASR bias. *C. picta* displays nesting plasticity in response to climate (Refsnider and Janzen 2012) and thus has the capcity to respond to ASR if biases are detected. Here, we test if adaptive sex allocation occurs in *C. picta* by observing nest timing, vegetation cover, and depth in groups with experimentally manipulated ASR. We predict that *C. picta* will display nesting behaviour that increases the chance of producing offspring of the rare sex. Specifically, we expect to observe nesting behaviour that results in warmer nests in a male‐biased group and cooler nests in a female‐biased group.

## 2 Methods

### 2.1 Study System

The painted turtle (*C. picta*) is distributed across North America, occurring in diverse freshwater ecosystems located in remote to human‐dominated landscapes (Ernst and Lovich 2009). As in most turtles, temperatures during the middle of embryonic development determine sex: cold temperatures produce male hatchlings and hot temperatures produce female hatchlings, with a pivotal temperature of ~28°C in our source population (Janzen 1994a; Carter et al. 2019). There is considerable variation in the adult sex ratio between populations in this species, with ASRs from 1:3 to 3:1 males to females reported (Ernst 1971; Bayless 1975; Freedberg and Bowne 2006; Hughes 2011; Dupuis‐Désormeaux et al. 2017; Vanek and Glowacki 2019). Climate‐change models predict that anthropogenically‐induced warming could further skew sex ratios in this species (Refsnider and Janzen 2016). Other factors, such as sex‐specific mortality from automobile traffic or predation, xenobiotic oestrogen exposure, and sex‐biased collecting (Pritchard and Trebbau 1984; Aresco 2005; Kohno 2021), have documented effects on reptile sex ratios and may collectively influence the ASR of *C. picta* populations (Schwanz et al. 2010).

The average age at first reproduction for *C. picta* increases with latitude (Ernst and Lovich 2009); in Illinois, the mean age/plastron length at maturity is 3–4 years/11.6 cm for males and 4–6 years/15.1 cm for females (Moll 1973). Despite this extended time to maturity, the offspring sex ratio influences the future recruitment of females to the breeding population in our source population (Schwanz et al. 2010). Offspring sex ratios vary considerably and are frequently male‐biased at our collection site, suggesting that adults may experience inter‐annual variation in sex ratios and biased ASRs (Schwanz et al. 2010). In our Illinois population, the maximum life expectancy (mean cumulative survival < 0.05%) is estimated to be 27 years for males and 32 years for females (Reinke et al. 2020). Consequently, generational overlap may reduce, but not eliminate, selection for parental responses to ASR bias (Schwanz, Janzen, and Proulx 2010). Therefore, there is potential for sex‐ratio selection to act on mechanisms of sex allocation in response to ASR.

Adult female painted turtles conceivably have the opportunity and means for gauging the number of adult males in their vicinity, both in the wild and in our experimental enclosures (described below). The home range of *C. picta* likely depends on the environmental features of a particular locality (Ernst and Lovich 2009); however, multiple studies of movement patterns suggest that home ranges of both males and females greatly exceed the area of our experimental ponds (McCulloch and Secoy 1983; Ernst and Lovich 2009; Rowe and Dalgarn 2010). In one population of *C. picta*, males and females do not generally differ in their use of space and resources within their home ranges (Rowe 2003), suggesting habitat use is not spatially sex‐segregated in this species. Painted turtles bask communally and mixed sex‐basking aggregations have been observed (Lovich 1988).

ASR can influence animal social dynamics and have subsequent effects on mating systems (Székely, Weissing, and Komdeur 2014; Schacht et al. 2022), but the relationship between ASR and reproductive behaviour in painted turtles is not well understood. Breeding occurs over an extended period during the boreal summer and autumn (Gist, Michaelson, and Jones 1990), with courtship activities resuming in spring after hibernation (Ernst and Lovich 2009). Aquatic visual and tactile courtship displays performed before mating offer the potential for female assessment of male availability (Ernst and Lovich 2009). Males are not territorial in this species and may attempt to mate with as many females as encounter rates allow (Pearse and Avise 2001; Pearse, Janzen, and Avise 2002). Additionally, harassment of *C. picta* females by males has been documented (Moldowan, Brooks, and Litzgus 2020). Given the biology and life history of this species, it is likely that adult painted turtles in our Illinois population experience sex‐ratio bias, have the capacity to detect this bias, and would benefit from responding via manipulation of offspring sex ratios.

### 2.2 Experimental Design

Our experimental design is as described in Mitchell et al. (2017) and Judson et al. (in press). We constructed three outdoor experimental enclosures (25 × 55 × 1 m) surrounded with silt fencing and aluminium flashing at the Iowa State University (ISU) Horticulture Farm. Each enclosure included an aquatic (19 × 15 × 1.5 m) and terrestrial (948 m^2^) environment. This terrestrial environment consisted of a gently south‐sloping grassy area with a 2 × 3 matrix of artificial shade structures, each of which was roughly 3 m from any neighbouring shade structure. Shade structures were constructed from a 1.5 m^2^ piece of garden shade cloth atop a 1 m steel T‐post (see Figure S1 in Appendix S1).

We used hoop nets to capture adult painted turtles in July 2014 (after the nesting season) from the Thomson Causeway Recreation Area (TCRA) in Thomson, IL, located ~300 km east of the experimental pond complex at ISU. We determined that the turtles were sexually mature by assessing their size, sexually‐dimorphic characters (e.g., elongated foreclaws in males), and the annual growth rings on their plastron (Moll 1973; Hoekstra et al. 2018). We assigned individual IDs by marking turtles with unique combinations of notches on their marginal scutes. We transported turtles via automobile in covered plastic boxes to the experimental pond complex. In October 2014, we drained all ponds and transported turtles to ISU for overwintering with their pondmates. We simulated natural hibernation conditions in cold rooms (thermal regime: ~10°C in November and in March; ~4°C in December–February), where we housed turtles in bins filled with conditioned water and set lighting to mimic the local photoperiod from November to March.

In April of 2015, we seeded the ponds with adult turtles in differing sex ratios (~M:F: 3:1, 1:1, and 1:3), reflecting those documented in wild populations (Hughes 2011; Dupuis‐Désormeaux et al. 2017). We refer to these ASR treatments as: male‐biased (M>F), approximately equal (M=F), and female‐biased (M<F). To ensure prolonged exposure to the ASR treatment, we allowed turtles to acclimatise for one breeding season before measuring nest characteristics in 2016. Our 2016 sample sizes were: M>F: 23 males, seven females; M=F: 10 males, eight females; and M<F: four males, 11 females. Turtles remained in the ponds during the summer months, consuming aquatic plants, anurans, invertebrates that colonised the ponds, and supplementary Mazuri Aquatic Turtle Diet. During the winter months, turtles hibernated under the same conditions described for 2014.

### 2.3 Measurement of Nest Characteristics

In May and June of 2016, we monitored pond areas hourly for nesting activity from 0600 to 2200 h. After a turtle finished nesting, we noted her identity, recorded the date, and removed eggs to measure nest depth and for transport to the laboratory. We quantified South West canopy openness (from here on referred to as SW openness) over the nest via hemispherical photography and Gap Light Analysis software (Doody et al. 2006; Bodensteiner et al. 2023). SW openness was used because it is the directional aspect that best predicts nest temperature (Weisrock and Janzen 1999) and offspring sex ratio (Janzen 1994a), and because it is both repeatable (Delaney, Hoekstra, and Janzen 2020) and heritable (McGaugh et al. 2010). In total, 30 nests were laid across all ponds in 2016 (8 in M>F, 12 in M=F, and 10 in M<F). Eight of these nests were produced by females who laid a second clutch (1 in M>F, 4 in M=F, and 3 in M<F). We intended to assess whether females differentially allocated sex‐biasing factors (e.g., steroid hormones, per Bowden, Ewert, and Nelson 2000) to their clutch of eggs in response to the ASR treatments; however, a power failure during the period of gonadal differentiation caused nearly all offspring to have testes (201 male vs. 7 female offspring) and thereby greatly limited our capacity to assess any impacts of physiological maternal effects. Upon completion of the nesting season, we drained all experimental ponds, removed the turtles, and measured their straight carapace length (SCL). In early August, we returned the 63 healthy adults to the site of capture at the TCRA, as required by our permits.

### 2.4 Statistical Analysis

To determine if nest date, SW openness, and nest depth differed between the ASR treatments, we used the function ‘lm’ from base R to fit separate linear models with day of the year, SW openness, and nest depth as response variables, and ASR treatment and maternal SCL as predictor variables. We included maternal SCL in our model because nesting behaviour is linked to body size in *C. picta* (larger mothers construct deeper nests, Morjan 2003; Refsnider et al. 2014; Delaney, Hoekstra, and Janzen 2020), though maternal body size did not differ between the treatments (see Table 1 and Figure S2 in Appendix S1). We used the R package “DHARMa” to simulate residuals of the models in order to evaluate normality and homogeneity of variance (Hartig and Lohse 2022). We used the R package “emmeans” to extract model‐predicted treatment means and 95% confidence intervals and to conduct comparisons of nest characteristics between the experimental ponds (Lenth 2023). As our hypothesis is directional (mothers will select nest sites that promote the development of the rare sex), we tested whether nest‐site characteristics differed in the expected direction (i.e., would mean values from the M=F and M<F treatments produce cooler nests than the characteristics of nests in the M>F treatment?).

**TABLE 1:** One‐way comparison of model‐estimated mean lay dates.

Pond	Mean lay date	SE	df	Lower 95% CI	Upper 95% CI	*t*‐ratio	*p*
M = F	152 (May 31st)	2.02	24	148	156	−0.457	0.326
M < F	153 (June 2nd)	2.20	24	149	158	0.227	0.589

*Note:* We expect that, if mothers adaptively allocate offspring sex in response to ASR by varying nest timing, mothers in the M < F treatment should nest earliest, followed by the M = F treatment, and the M > F treatment, as early nesting leads to cooler nest temperatures, which would be required to produce additional males in this species. Thus, this test determines if the treatment mean lay date is earlier than the mean lay date of the male‐biased treatment (153 or June 1st).

Individual maternal variation may influence nest‐site characteristics; however, including maternal ID as a random effect in our analyses produced models with a singular fit (i.e., some elements of the variance–covariance matrix are equal to zero, Bates et al. 2015). Including maternal ID in the model may be problematic as singular models can have lower statistical power and produce less reliable parameter estimates, so we removed the term from our final models, as recommended by Matuschek et al. (2017).

All analyses were conducted in R (version 4.3.2, R Core Team 2023). Data visualisation was completed in R Studio using the “ggplot2”, “patchwork”, and “showtext” packages (Qiu 2023; Wickham 2016; Pedersen 2023).

## 3 Results

### 3.1 Nesting Date

**FIGURE 1:**
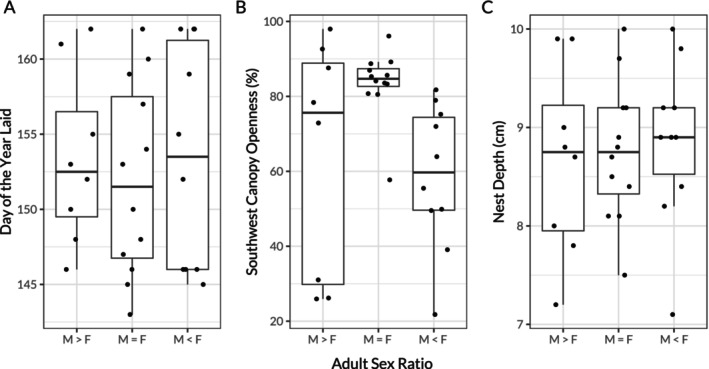
A comparison of nest characteristics between the three ASR treatments. A) Shows the day of the year that nests were laid, B) shows canopy oppeness over the Southwest half of the nests, and C) shows the depth of the nests.  M > F is the male‐biased treatment, M = F is the approximately equal treatment, and M < F is the female‐biased treatment. The boxplot shows five summary statistics: the median (central horizontal line), the 25% percentile (lower hinge), the 75% percentile (upper hinge), the smallest value within 1.5 × the interquartile range (lower whisker), and the largest value within 1.5 × the inter‐quartile range (upper whisker).

There were negligible differences between the mean lay date of nests in the three treatment ponds (Figure 1A). We found no association between lay dates and ASR treatment or maternal SCL (*p* = 0.983, *F*_5,24_ = 0.134, *R* ^2^ = 0.027, Table 1).

### 3.2 South West Canopy Openness

**FIGURE 2:**
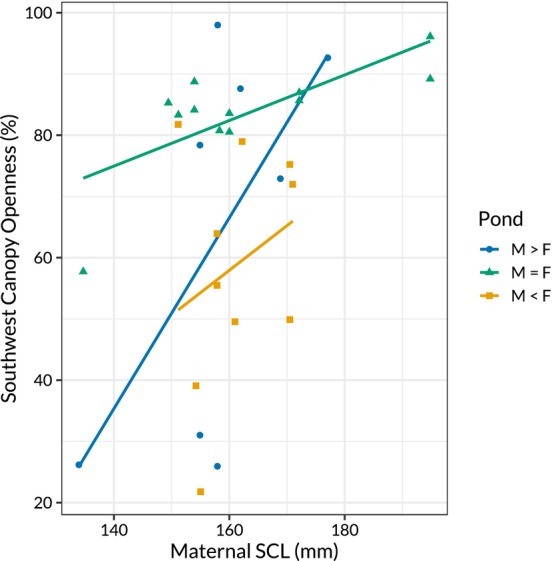
Relationship between maternal straight carapace length and southwest canopy openness over nest sites. Larger mothers selected more open nest sites, and this relationship was stronger in the biased sex‐ratio treatments than in the M = F pond.

Canopy openness of chosen nest sites varied between treatment ponds and was associated with maternal SCL (*p* = 0.005, *F*_5,24_ = 4.476, *R* ^2^ = 0.483, Figure 1B, Table 2). Nests laid in the M = F pond had greater canopy openness than in the M>F pond or the M<F pond (*p* = 0.048). However, the trend is unlikely to represent an adaptive response to ASR bias, as canopy openness did not differ in a direction that would produce nest temperatures that would promote the development of the rare sex (Table 2). Additionally, there was a marginally non‐significant interacting effect of pond treatment and maternal SCL on canopy openness, where SCL was less strongly associated with canopy openness in the M=F pond in comparison to the other experimental treatments (Figure 2). On average across the treatments, a 1 mm increase in maternal SCL was associated with a 1.56% increase in canopy openness (*p* = 0.008), but in the M=F pond, a 1 mm increase in maternal SCL was associated with only a 0.37% increase in canopy openness (*p* = 0.067). Although mean maternal SCL was similar between the treatment ponds, the M=F pond was substantially more variable (M>F: mean = 158 mm, SD = 12.5; M=F: mean = 163 mm, SD = 17.9; M<F: mean = 161 mm, SD = 7.3, see Figure S2 and Table S1 in Appendix S1). Additionally, the three largest females in the F=M treatment nested twice, which may drive the different trend in SCL versus canopy openness, as this relationship appears to be weaker for larger females (Figure 2).

**TABLE 2:** One‐way comparison of model‐predicted mean South West canopy openness (%).

Pond	Mean canopy openness (%)	SE	df	Lower 95% CI	Upper 95% CI	*t*‐ratio	*p*
M = F	82.8	5.18	24	72.1	93.5	2.803	0.995
M < F	58.8	5.64	24	47.1	70.4	−1.690	0.052

*Note:* We expect that if mothers adaptively allocate offspring sex by varying vegetation cover over the nest, then mothers in the M < F treatment will choose the least open canopies, followed by the M = F treatment, and the M > F treatment, as decreased canopy openness leads to cooler, male‐producing nests. Accordingly, this test determines if the treatment mean canopy openness is lower than the mean canopy openness of the male‐biased treatment (68.3%).

### 3.3 Nest Depth

We found no difference in mean nest depth between the three treatment ponds and no effect of maternal SCL on mean nest depth (*p* = 0.931, *F*_5,24_ = 0.260, *R* ^2^ = 0.051, Figure 1C, Table 3).

**TABLE 3:** One‐way comparison of model‐predicted mean nest depth (mm).

Pond	Mean nest depth	SE	df	Lower 95% CI	Upper 95% CI	*T*‐ratio	*p*
M = F	8.75	0.247	24	8.24	9.26	0.354	0.363
M < F	8.86	0.269	24	8.30	9.42	0.744	0.232

*Note:* If mothers adaptively allocate offspring sex by varying nest depth, we expect that mothers in the M > F treatment will construct the shallowest nests, followed by the M = F treatment, and the M < F treatment, as deeper nests are cooler and produce more males. Thus, this test determines if the treatment mean nest depth is lower than the mean nest depth of the male‐biased treatment (8.66 mm).

## 4 Discussion

In this investigation, we assessed the capacity of female painted turtles to influence offspring sex in response to variation in the adult sex ratio of their experimental population. We found no evidence that individuals adjusted their nesting behaviour to produce offspring of the rare sex. Our results suggest that painted turtles do not exhibit adaptive behavioural plasticity in response to ASR. If adaptive sex allocation occurs in this species, it may be in response to cues other than ASR, such as the climate during the nesting season (Schwanz and Janzen 2008). Alternatively, *C. picta* may utilise physiological mechanisms of control over offspring sex instead of behavioural adaptations (e.g., Bowden, Ewert, and Nelson 2000).

Sex allocation theory predicts that reproductive behaviour should respond to biased sex ratios (Werren and Charnov 1978). However, evidence of adaptive sex allocation in response to the sex ratio of demographic sub‐groups (juveniles, adults, or breeding individuals) is mixed (e.g., for: McLain and Marsh 1990; Olsson and Shine 2001; Michler et al. 2013; Benvenuti et al. 2018; Brooksmythe et al. 2018; Firman 2020; Arrieta et al. 2024; against: Bensch et al. 1999; Wheelwright and Seabury 2003; Le Galliard, Fitze, Cote, et al. 2005; Allsop et al. 2006; Perlut et al. 2014). It is possible that differences in species' capacity to respond to ASR are due to variation in the reliability of cues for ASR in different taxa. In some species, a biased ASR results in increased competition for mates between members of the more abundant sex and increased mating attempts directed at members of the rare sex, cues of ASR that individuals may detect and consequently alter their behaviour (Krupa and Sih 1993; Clutton‐Brock and Parker 1995; Sztatecsny et al. 2006; Le Galliard, Fitze, Ferrière, et al. 2005; Le Galliard, Cote, and Fitze 2008; Grayson et al. 2012; Sanmartín‐Villar, Yu, and Cordero‐Rivera 2023; Schacht et al. 2022). However, this does not guarantee that such cues are present and detectable across taxa. While potential cues for *C. picta* ASR exist (Ernst and Lovich 2009; Moldowan, Brooks, and Litzgus 2020; see ‘Study System’ for summary), it is possible that females cannot interpret these cues or that high male density, in addition to ASR bias, is required to elicit an adaptive behavioural response. Furthermore, female painted turtles may require prologued exposure to cues of ASR bias to initiate a shift in nesting behaviour. In our study, we exposed females to 2 years of experimentally manipulated sex ratios; however, it is possible that behavioural responses may occur over longer time periods.

Alternatively, it is possible that reptiles with TSD respond directly to the thermal conditions they experience before and during the nesting season rather than to ASR. Variation in nest phenology, sitechoice, and construction in response to climate has been detected in reptiles with TSD, including *C. picta* (Morjan 2003; Ewert, Lang, and Nelson 2005; Schwanz and Janzen 2008; Refsnider and Janzen 2012; Refsnider et al. 2014). As ectotherms, behavioural plasticity in response to temperature is a key aspect of reptile biology that requires reptiles to assess thermal opportunities in their environment (Angilletta 2009). Thus, reptiles may rely on their perception of the thermal environment to detect climate conditions that could result in biased offspring sex ratios rather than responding to ASR. Additionally, environmental variability may constrain adaptive sex allocation (West and Sheldon 2002). The reliability of seasonal climate vs. ASR for predicting the social environment offspring will experience as adults could vary depending on climate and life history factors. Current ASR is a reliable predictor of future ASR if the climate is relatively stable or if annual climate fluctuations occur around a constant mean (Schwanz et al. 2010). However, seasonal climate may be a better predictor of future ASR if the climate is undergoing directional change. Furthermore, selection on offspring sex ratios may be reduced by overlapping generations in long‐lived species, such as *C. picta*, as intra‐generational sex‐ratio bias is ameliorated by generational interbreeding (Schwanz et al. 2009; Schwanz, Janzen, and Proulx 2010). In such life histories, sex allocation may be driven by sex‐specific fitness effects of incubation temperature (Charnov and Bull 1977; Schwanz and Georges 2021), rather than balancing annual offspring sex ratios.

Sex‐ratio selection is one of many factors that can influence nest‐site choice (Refsnider and Janzen 2010). We may have failed to observe adaptive manipulation of sex ratios because other factors took precedence in influencing *C. picta* nesting behaviour. As incubation temperature influences offspring survival, hard selection (i.e., fitness is independent of the phenotypes of conspecifics; see Bell et al. 2021) for a nest site that promotes survival may be stronger than soft selection for the rare sex (Schwarzkopf and Brooks 1987; Morjan 2003; Noble et al. 2021; but see Mitchell, Maciel, and Janzen 2013). Reptiles select oviposition sites with thermal conditions that promote offspring survival (Weisrock and Janzen 1999; Leslie and Spotila 2001; Ewert, Lang, and Nelson 2005), which may differ from the conditions that allow adaptive allocation of clutch sex ratios (Refsnider and Janzen 2010). Additionally, maternal condition may influence nest‐site choice. Turtle mothers expend significant energy travelling to the nest location and constructing the nest, during which they are particularly vulnerable to predation (Tucker, Filoramo, and Janzen 1999; Delaney, Janzen, and Warner 2017; Marchand, Le Gal, and Georges 2021). Younger females and those in poor condition may not have the option to select a site that promotes the production of the rare sex, instead selecting safer or more accessible nest sites (Harms et al. 2005; Delaney and Janzen 2020; but see Refsnider et al. 2015). Age‐ and condition‐dependent nest‐site choice may explain our finding that maternal body size, a proxy for age in *C. picta*, predicted nest canopy cover. Because heritability of nest‐site choice is modest and conditional on the environment (McGaugh et al. 2010, 2011; McGaugh and Janzen 2011), evolutionary change resulting from weak sex‐ratio selection may be overshadowed by strong selection for phenotypes that promote maternal and offspring survival.

To respond to a biased ASR and adaptively manipulate offspring sex via nesting behaviour, suitable nest sites must be available. Extreme weather conditions may restrict nest‐site choice, as the range of nests with temperatures promoting offspring survival and development may be reduced. For example, preferences for sites with high shade cover (i.e., cooler sites) may override preferences for sites with cover levels that would balance sex ratios if there is a risk of embryo death due to extreme temperatures at those sites. Our study was conducted during an unusually hot nesting season. In particular, June 2016 was the 12th hottest June out of 129 June records at the time of publishing (National Oceanic and Atmospheric Association (NOAA) 2023). Individuals in different ASR treatments may have behaved similarly because high temperatures caused nest sites that would normally be optimal for offspring sex ratio to become unsuitable for nesting, constraining the expression of nesting preferences. A study on our source population that estimated sex ratios and survival across a range of annual climates found that the temperature of maternally‐selected nest sites would not improve offspring survival in comparison to randomly placed artificial nests but would be more likely to produce a 1:1 sex ratio than a random site (Mitchell, Maciel, and Janzen 2013). Using those results, we calculated that similarity in the predicted annual average temperatures of maternal and random nests was associated with an increase in sex‐ratio bias in maternal nests, suggesting that the capacity of mothers to nest differently from random is reduced under extreme climates (see Figure S3 in Appendix S1). Taken together, these results suggest that sex‐ratio selection may influence nest‐site choice but that extreme climates reduce the capacity of mothers to effectively express nest preferences (sensu Janzen 1994a). This finding is cause for concern, as extreme weather conditions are increasing as the climate warms (Robinson 2012) and could already be hampering nest‐site choice. While sex‐ratio selection can drive local adaptation of nesting behaviour in TSD species (Bodensteiner et al. 2023), it is likely that these processes occur too slowly to allow long‐lived species, such as *C. picta*, to adapt to rapid climate change without the capacity to express plastic responses to sex ratio bias (Janzen 1994b; Mitchell and Janzen 2010).

Nesting individuals must be able to recognise nest characteristics that would promote the development of the rare sex in order to take advantage of those opportunities. There is significant evidence that natural canopy cover influences nest‐site choice in *C. picta* and other reptiles (Morjan 2003; Angilletta, Sears, and Pringle 2009; Mitchell and Walls 2013; Mitchell, Maciel, and Janzen 2013; Jackson et al. 2019; Bodensteiner et al. 2023), though no study has investigated whether reptiles respond similarly to artificial shade. Snapping turtles (*Chelydra serpentina*) select similar nest microhabitats in natural and human‐modified landscapes, which suggests that this species is able to interpret cues from anthropogenic structures (Kolbe and Janzen 2002). However, we cannot be certain that nesting turtles perceive artificial shade installations as similar to natural vegetation cover. It is possible that the turtles in our study did not display differences in nesting behaviour in response to ASR because they could not detect nesting opportunities—despite the presence of artificial shade in their pond enclosures—that would allow them to adaptively influence the sex of their offspring. Future research addressing this question would provide valuable information about human‐reptile interactions and inform the implementation of artificial nest shading as a conservation strategy.

Finally, turtles with TSD might adaptively influence offspring sex via a means other than nesting behaviour. Rather than manipulating the nest environment to produce hatchlings of the rare sex, mothers may influence embryonic sexual development in response to temperature. For example, maternally‐derived yolk hormones affect offspring sex in turtles with TSD (Bowden and Paitz 2018). However, it is not known whether females possess mechanisms to adaptively allocate sex‐influencing compounds to eggs. We sought to explore the potential impact of maternal physiology on offspring sex; however, an unexpected incubator power failure eliminated that opportunity. Examining the influence of ASR on maternal reproductive physiology could prove a fruitful direction for future research.

Our study is limited by a lack of replication at the treatment level due to logistical and permitting constraints that prevented the construction of more than one experimental pond per sex ratio treatment. This experiment would require an additional 63 turtles per full replicate in order to keep the group sexratio and female density consistent across the treatments. As the turtles must be sampled from the same source population and remain in experimental housing for at least 12 months, this would put an unjustified burden on the sampled population. Thus, it is possible that variation in nesting opportunities between the ponds obscured the expression of different nesting behaviour between the treatments. However, we took precautions to ensure that canopy cover, an important variable governing nesting behaviour in our study species (Bodensteiner et al. 2023), was similar between the three treatment ponds. The results from each pond are highly similar (Figure 1), so it is unlikely that minor differences between the ponds are obscuring substantive differences in nesting behaviour. Nevertheless, the lack of variation in canopy cover in the M = F treatment is notable (Figure 1). It is possible that canopy cover combined with another, unidentified aspect of nest site choice (e.g., substrate type), leading to the appearance of highly specific canopy cover preferences in this treatment. This result highlights the need to further understand nest site selection in *C. picta* and other species with environmentally sensitive development.

While adaptive plasticity can confer significant fitness advantages, it relies on the availability of accurate information about the environment and the capacity to respond to that information (Snell‐Rood and Ehlman 2021). Adaptive sex allocation is no exception, and while it is theoretically sound that parents should produce offspring of the rare sex, individuals may not have the capacity to detect or respond to cues of ASR. Alternatively, responses may be overshadowed by other selective pressures or only be detectable under certain environmental conditions. Few studies have attempted to assess adaptive sex allocation in vertebrates in response to experimentally manipulated adult sexual ratios. While these investigations are resource‐demanding and logistically complex, it is essential that we understand how species will respond to sex‐ratio disruption if we wish to mitigate the risk of extinction associated with anthropogenically‐induced demographic shifts.

## Author Contributions

**Claudia Crowther:** formal analysis (lead), investigation (lead), visualization (lead), writing – original draft (lead), writing – review and editing (lead). **Clare I. M. Adams:** investigation (equal), writing – review and editing (supporting). **Andy Fondren:** investigation (equal), writing – review and editing (supporting). **Fredric J. Janzen:** conceptualization (lead), formal analysis (supporting), funding acquisition (lead), investigation (equal), supervision (lead), writing – review and editing (supporting).

## Ethics Statement

The following research methods were approved by Iowa State University IACUC (12‐03‐5570‐J and 12‐03‐5566‐J). Permits for painted turtle collection were provided by the United States Army Corps of Engineers, the United States Fish and Wildlife Service (SUP 32576‐028), and the Illinois Department of Natural Resources (NH14.0073).

## Conflicts of Interest

The authors declare no conflicts of interest.

## Supporting information


Appendix S1:

## Data Availability

The data that support the findings of this study are openly available in Dryad at http://doi.org/10.5061/dryad.vt4b8gv20.
