# Supplementary material for: Adult Sex‐Ratio Bias Does Not Lead to Detectable Adaptive Offspring Sex Allocation Via Nest‐Site Choice in a Turtle With Temperature‐Dependent Sex Determination
Source: Ecol Evol. 2024 Nov 13;14(11):e70543. doi: 10.1002/ece3.70543 (PMC11560344; doi:10.1002/ece3.70543)
Supplement: Supplementary file 1 — Appendix S1: [file ECE3-14-e70543-s001.docx]

**Appendix**

B

A


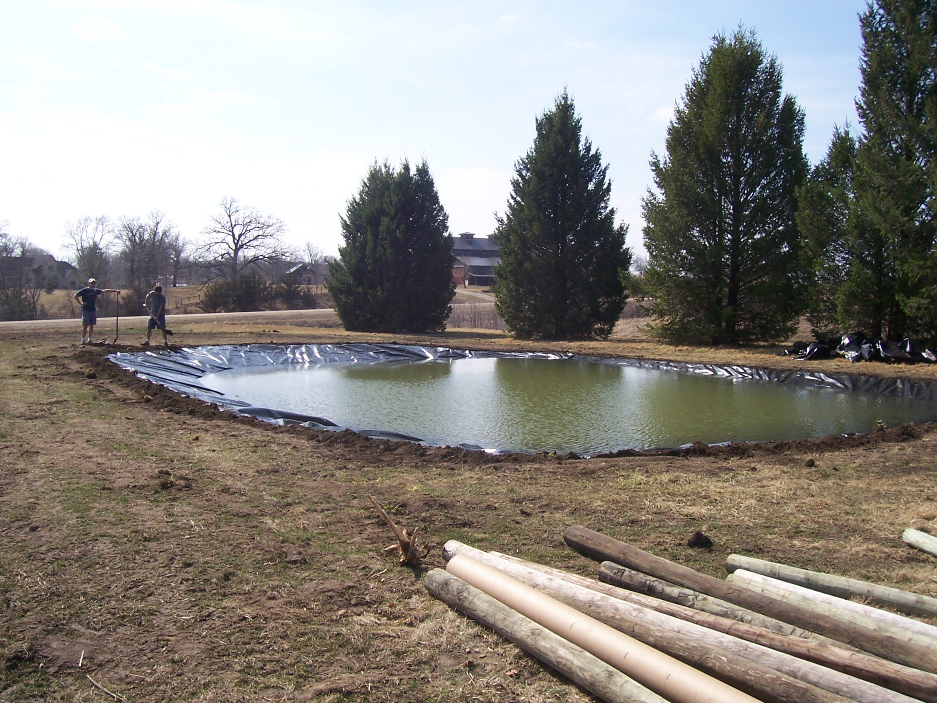

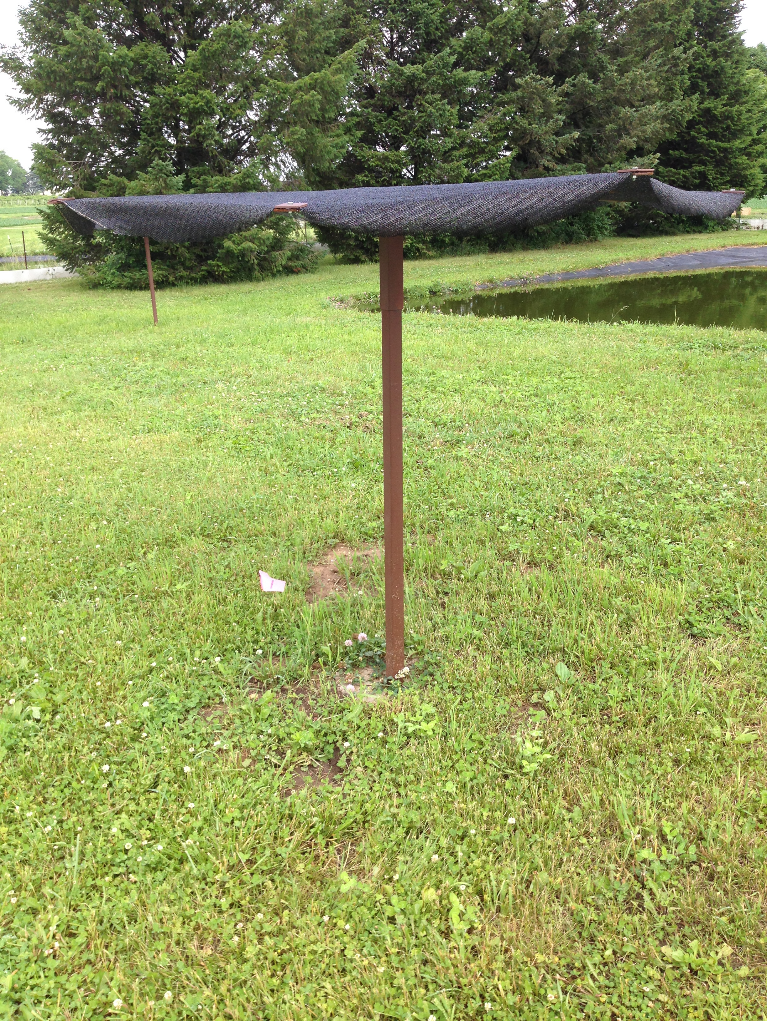


**Figure S1.** Construction of the experimental enclosures. S1. A) shows the aquatic area of an enclosure and S1. B) shows an example of the artificial shade structures installed in the enclosures, with a pink flag denoting the location of a turtle nest.

**Comparing Maternal SCL Across Treatments**

We fit a linear model to test if maternal SCL varied between the experimental ponds. We used the maternal SCL (mm) for each nest, so mothers that constructed two nests are included twice in the analysis, in order to account for any maternal size effects on clutch number. Maternal SCL did not differ between the treatments (P = 0.774, F_2,27_ = 0.258, R^2^ = 0.019). However, because the variability of SCL differed between the treatment groups (the standard deviation of SCL measures for the M > F, M = F, and M < F were 12.5 mm, 17.9 mm, and 7.3 mm respectively), we included SCL as a predictor variable in our analyses to account for these differences in variance.


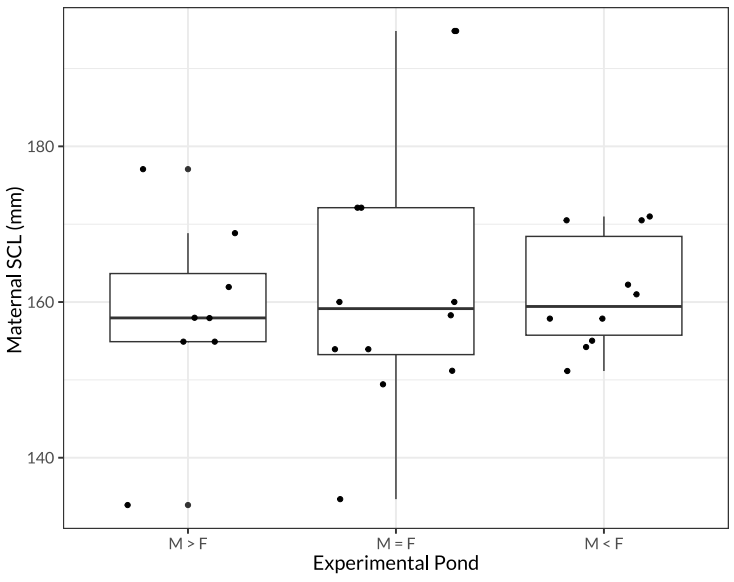


**Figure S2.** Comparison of straight carapace lengths (mm) of individual females in the three experimental ponds.

**Table S1.** Results of a Tukey honest significant differences test comparing maternal straight carapace length (mm) between the experimental treatments. We found no difference in SCL measures between the treatments.

| Contrast | Estimated Difference (mm) | Lower 95% CI | Upper 95% CI | p-value |
| --- | --- | --- | --- | --- |
| M=F vs. M>F | 4.51 | -11.04 | 20.06 | 0.755 |
| M<F vs. M>F | 2.70 | -13.46 | 18.85 | 0.910 |
| M<F vs. M=F | -1.81 | -16.40 | 12.77 | 0.949 |


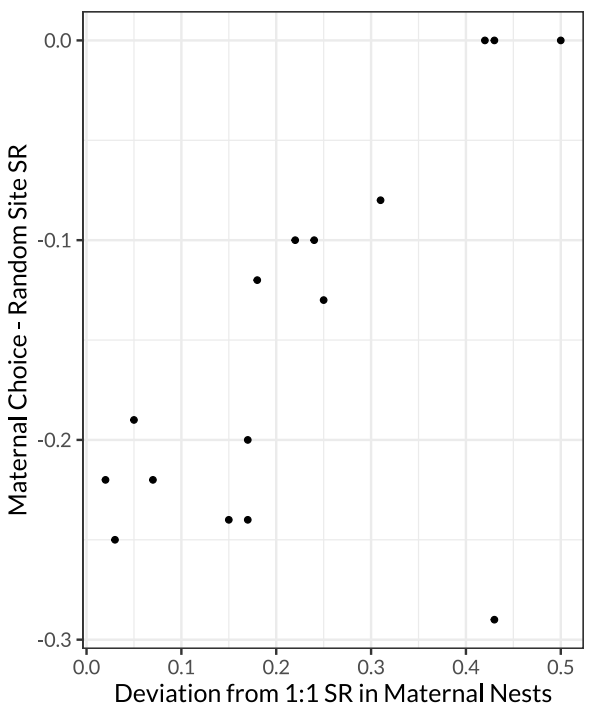


**Figure S3.** The findings of Mitchell et al. (2013) demonstrate an association between clutch sex ratio bias and similarity in the sex ratios produced by maternal and randomly chosen nest sites. The x-axis shows the predicted annual average deviation from a 1:1 clutch sex ratio in maternally-selected nest sites (|SR - 0.5|). The y-axis shows the predicted annual average difference in clutch sex ratios between maternal and random nest sites.
